# Use of Intranasal Oxytocin to Treat Adult Autism Spectrum Disorder: A Randomized Double Blind Controlled Trial

**DOI:** 10.1192/j.eurpsy.2024.151

**Published:** 2024-08-27

**Authors:** S. Faraji Niri, J. Alaghband-rad, M. Motamed, S. A. Hojjati, S. Seyed Alipour, N. Mohebbi

**Affiliations:** ^1^Psychiatry, Tehran University of Medical Sciences; ^2^Urology, Shahid Beheshti University of Medical Sciences; ^3^Pharmacology, Tehran University of Medical Sciences, Tehran, Iran, Islamic Republic Of

## Abstract

**Introduction:**

Autism Spectrum Disorder (ASD) is characterized by impairments in social interaction and restricted interests. It has been reported that oxytocin may improve processing of social cues and emotions in adults with ASD.

**Objectives:**

The aim of this study was to evaluate the therapeutic effects and safety of intranasal oxytocin in this population.

**Methods:**

Thirty-nine patients with ASD were randomly assigned to two groups: one group received intranasal oxytocin and the other group received a placebo, with 24 units administered every 12 hours for 8 weeks. The patients were evaluated using the Autism Quotient (AQ), Ritvo Autism Asperger Diagnostic Scale – Revised (RAADS-R), Social Responsiveness Scale (SRS), Clinical Global Impression (CGI), and World Health Organization Quality of Life-BREF (WHOQL-BREF) questionnaires at weeks 0, 4, and 8.

**Results:**

The intervention group showed clinical improvements in RAADS-R (P=0.010), social communication subscale of SRS (P=0.002), CGI (P=0.000), physical (P=0.004), psychological (P=0.006), and social relationships (P=0.046) domains of WHOQL-BREF. Improvements reached their maximum at week 4 and were maintained until week 8 (Table 1).

**Table 1.** Effect of group, time time-group interaction and the effect size

	Time			Group			Time-Group Interaction		
	F	P-Value	Effect Size (Partial Eta Squared)	F	P-Value	Effect Size (Partial Eta Squared)	F	P-Value	Effect Size (Partial Eta Squared)
**AQ**	19.44	**0.000**	0.344	0.391	0.536	0.01	2.63	0.079	0.066
**RAADS-R **	12.68	**0.000**	0.255	0.944	0.338	0.025	7.250	**0.001**	**0.164**
**SRS**	23.63	**0.000**	0.390	0.050	0.823	0.001	7.82	**0.001**	**0.175**
**WHOQL-BREF -Physical Health**	6.34	**0.003**	0.146	0.115	0.737	0.003	5.7	**0.005**	**0.134**
**WHOQL-BREF -Psychological Health**	8.31	**0.001**	0.183	0.048	0.828	0.001	6.14	**0.003**	**0.142**
**WHOQL-BREF -Social Relationships**	7.72	**0.001**	0.173	1.052	0.312	0.028	3.64	**0.031**	0.090
**WHOQL-BREF -Environmental Health**	4.87	**0.010**	0.116	0.162	0.690	0.004	2.69	0.074	0.068
**CGI**	22.08	**0.000**	0.374	2.28	0.139	0.058	9.42	**0.004**	0.203

AQ : Autism Spectrum Quotient, SRS : Social Responsiveness Scale, SCI : Social Communication Interaction, RRB : Restricted interest and repetitive behavior, WHOQL-BREF : World Health Organization Quality of life-BREF, CGI : Clinical Global Impression

**Conclusions:**

The findings of this study suggest that nasal oxytocin therapy can significantly improve social skills and quality of life in individuals with ASD. Further research is needed to determine the timing and scope of oxytocin’s effects across the lifespan.

**Disclosure of Interest:**

None Declared

